# Characteristics of patients initiated on edoxaban in Europe: baseline data from edoxaban treatment in routine clinical practice for patients with atrial fibrillation (AF) in Europe (ETNA-AF-Europe)

**DOI:** 10.1186/s12872-019-1144-x

**Published:** 2019-07-12

**Authors:** Raffaele De Caterina, Peter Kelly, Pedro Monteiro, Jean Claude Deharo, Carlo de Asmundis, Esteban López-de-Sá, Thomas W. Weiss, Johannes Waltenberger, Jan Steffel, Joris R. de Groot, Pierre Levy, Ameet Bakhai, Wolfgang Zierhut, Petra Laeis, Michael Kerschnitzki, Paul-Egbert Reimitz, Paulus Kirchhof, on behalf of the ETNA-AF-Europe investigators

**Affiliations:** 1 Università degli Studi di Pisa, Pisa, Italy; 2 HRB Stroke Clinical Trials Network Ireland, University College Dublin, Dublin, Ireland; 3 Centro Hospitalar e Universitário de Coimbra, Coimbra, Portugal; 4 Hôpital de la Timone, Marseille, France; 5 Universitair Ziekenhuis Brussels, Dilbeek, Belgium; 6 Hospital Universitario La Paz, IDIPAZ, Madrid, Spain; 7 Karl Landsteiner Institute for Cardiometabolics and SFU, Vienna, Austria; 8 University of Munster, Münster, Germany; 9 University Hospital of Zurich, Zürich, Switzerland; 10 Amsterdam University Medical Centers/University of Amsterdam, Amsterdam, The Netherlands; 11 Université Paris-Dauphine, PSL Research University, Paris, France; 12 Royal Free London NHS Foundation Trust, Chase Farm Hospital, London, UK; 13 Daiichi Sankyo Europe GmbH, Munich, Germany; 14 Institute of Cardiovascular Sciences, University of Birmingham, SWBH and UHB NHS Trusts, IBR 136, Wolfson Drive, Birmingham, B15 2TT UK; 15 The Atrial Fibrillation NETwork (AFNET), Münster, Germany

**Keywords:** Non-vitamin K antagonist oral anticoagulants, Real-world, Registry, Stroke prevention, Major bleeding, Safety outcomes

## Abstract

**Background:**

Non-vitamin K antagonist (VKA) oral anticoagulants (NOACs) have substantially improved anticoagulation therapy for prevention of stroke and systemic embolism in patients with atrial fibrillation (AF). The available routine care data have demonstrated the safety of different NOACs; however, such data for edoxaban are scarce. Here, we report baseline characteristics of 13,638 edoxaban-treated patients with AF enrolled between November 2016 and February 2018.

**Methods:**

ETNA-AF-Europe is a multinational, multi-centre, post-authorisation, observational study conducted in 825 sites in 10 European countries. Patients will be followed up for four years.

**Results:**

Overall, 13,980 patients were enrolled of which 342 patients were excluded from the analysis. Mean patient age was 73.6 years with an average creatinine clearance of 69.4 mL/min. 56.6% were male. The calculated CHA_2_DS_2_-VASc and HAS-BLED mean scores were 3.1 and 2.6, respectively. Overall, 3.3, 14.6 and 82.0% of patients had low (CHA_2_DS_2_-VASc = 0), intermediate (CHA_2_DS_2_-VASc = 1) and high (CHA_2_DS_2_-VASc≥2) risks of stroke, respectively. High-risk patients (those with prior stroke, prior major bleeding, prior intracranial bleed or CHA_2_DS_2_-VASc ≥4) comprised 38.4% of the overall population. For 75.1% of patients edoxaban was their first anticoagulant prescription, whilst 16.9% switched from a VKA and 8.0% from another NOAC. A total of 23.4% of patients in ETNA-AF-Europe received the reduced dose of edoxaban 30 mg. Overall, 83.8% of patients received an edoxaban dose in line with the criteria outlined in the label.

**Conclusion:**

Edoxaban was predominantly initiated in older, often anticoagulation-naïve, unselected European patients with AF, with a good overall adherence to the approved label.

**Trial registration:**

NCT02944019; Date of registration: October 24, 2016.

## Background

Atrial fibrillation (AF) is the most prevalent sustained cardiac arrhythmia, and a major cause of ischaemic stroke and disabilities. AF is associated with increased mortality and poses a high burden upon healthcare resources [1]. The risk of strokes related to AF can be substantially reduced through effective anticoagulation. In recent years, non-vitamin K antagonist (VKA) oral anticoagulants (NOACs) including the direct thrombin inhibitor dabigatran and the direct factor Xa inhibitors rivaroxaban, apixaban, and edoxaban, have greatly improved anticoagulation therapy for the prevention of stroke and systemic embolism in patients with AF. Randomised controlled trials (RCTs) have shown that these agents have at least similar efficacy compared to VKAs, with improved safety, specifically with regards to intracranial haemorrhage [2–6]. In the light of these findings, the European Society of Cardiology 2016 guidelines recommend using NOACs in preference to VKAs when oral anticoagulation is initiated in a patient eligible for a NOAC [7].

Edoxaban, a highly selective, once-daily, direct, reversible inhibitor of Factor Xa was approved for stroke prevention in patients with AF in Europe in 2015, as well as for the treatment and secondary prevention of venous thromboembolism (VTE) in adults. The recommended edoxaban dose is 60 mg once daily with a reduced dose of 30 mg once daily for patients with moderate or severe renal impairment (creatinine clearance 15–50 mL/min), low body weight (≤60 kg), or concomitant use of strong p-glycoprotein inhibitors, including cyclosporine, dronedarone, erythromycin, or ketoconazole [8].

The prescribing of NOACs has increased substantially over the last few years. Recent, real world evidence (RWE) studies have been enrolling patients with AF into various registries such as GLORIA-AF [9], ORBIT AF-II [10], XANTUS [11], GARFIELD-AF [12], DRESDEN NOAC Registry [13], PREFER in AF [14], EORP-AF Registry [15], Danish Registry [16] and retrospective claims analyses [17–20]. These have demonstrated the safety of different NOACs in routine clinical use. Since edoxaban was the last of the four NOACs to enter the market, published data on the use of edoxaban in routine clinical practice are still limited compared with other NOACs [16]. The objective of this analysis was to describe the characteristics of unselected European patients with AF initiated on edoxaban, to compare these characteristics with the European patients enrolled into ENGAGE AF-TIMI 48, and to describe adherence to the dosing recommendations for edoxaban.

## Methods

The Edoxaban Treatment in Routine Clinical Practice for Patients With Non Valvular Atrial Fibrillation (ETNA-AF-Europe) was designed as part of the risk management plan of edoxaban in order to assess the risks and benefits of the drug in routine care in unselected European patients with AF. ETNA-AF-Europe is part of the global ETNA initiative, which is composed of separate, non-interventional prospective ETNA-AF registries in Europe, East Asia, Brazil and Japan. The final ETNA-AF-Europe protocol was developed based on discussions with, and finally approved by the Pharmacovigilance Risk Assessment Committee (PRAC) of the European Medicines Agency. The primary objective of ETNA-AF Europe is to assess the safety of edoxaban by evaluating bleeding events, including intracranial haemorrhage; drug related adverse events, such as liver adverse events; and cardiovascular (CV) as well as all-cause mortality in routine care patients with AF treated with edoxaban up to 4 years, with regard to onset (relative to treatment with edoxaban) of the event, duration, severity and outcomes.

**Fig. 1:**
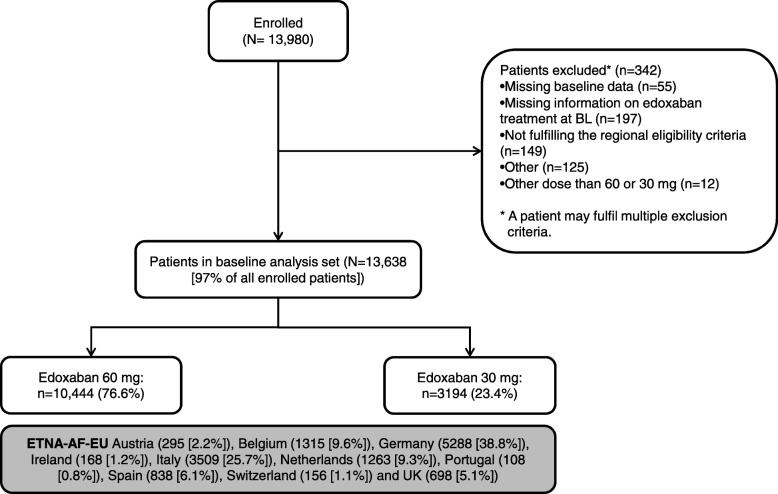
Overview of the ETNA-AF-Europe registry. *Some patients fulfilled more than one exclusion criteria

Details of the design of ETNA-AF-Europe including the statistical rationale have been published [21]. In short, ETNA-AF-Europe is a multinational, multi-centre, post-authorisation, observational study (Clinicaltrials.gov: NCT02944019) conducted in 825 sites (with at least one patient enrolled) in 10 European countries. All patients with non-valvular AF treated with edoxaban according to the summary of product characteristics (SmPC), could participate in the study with prior provision of written informed consent and no simultaneous participation in an interventional trial. No explicit exclusion criteria were defined. Over a period of 3 years, ETNA-AF-Europe enrolled 13,980 patients with AF confirmed within the last 12 months before enrolment. AF had to be confirmed by the investigators by electrical tracing (e.g., ECG, Holter monitoring, pacemaker or other implantable device). Detailed information on AF history and diagnosis, and on previous AF-related therapies was collected, including former anticoagulant treatment with VKAs, NOACs or heparins; previous or current antiplatelet drugs, antiarrhythmic and rate-control drugs and other therapies. The CHA_2_DS_2_-VASc and HAS-BLED scores were both reported by the investigators as well as calculated based on the baseline clinical characteristics of the patients. For the analysis here reported, calculated scores are used. Specific subgroup analyses are planned by edoxaban dose, patient age and country. Figure 1 provides an overview of the patient disposition in ETNA-AF-Europe. Of 13,980 enrolled patients, 13,638 were included in the baseline analysis set. Patients are to be followed up once a year for a total of 4 years.

The patient baseline characteristics from ENGAGE AF-TIMI 48 are used as an external comparator to the baseline data collected in ETNA-AF-Europe to better understand how the usage of edoxaban in routine clinical practice reflects on the trial setting in which edoxaban was tested. The ENGAGE AF-TIMI 48 cohort used for this purpose includes patients from only those European countries that are also participating in the ETNA-AF-Europe registry.

## Results

### Baseline characteristics

**Fig. 2:**
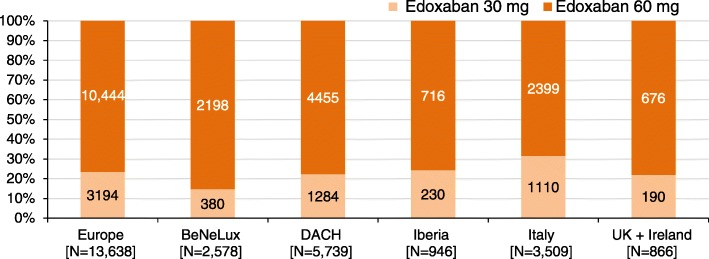
Number of patients per region (by dose) in the European ETNA-AF registry. BeNeLux, Belgium, the Netherlands, and Luxembourg; DACH, Germany, Austria, Switzerland

A total of 13,980 patients were enrolled between November 2016 and February 2018 of which 342 patients were excluded from the analysis because they were missing baseline data, missing information on edoxaban treatment at baseline, not fulfilling the regional eligibility criteria, receiving doses other than 60 or 30 mg and other reasons (Fig. 1). Of those, 13,638 patients were evaluated in the analysis of demographics and other baseline characteristics (Fig. 1). The baseline demographics and clinical characteristics of the entire analysis set as well as for the edoxaban 60 mg and 30 mg dose groups separately, are summarised in Table 1. Per-country split of the number of patients was as follows: Austria (*n* = 295 [2.2%]), Belgium (*n* = 1315 [9.6%]), Germany (*n* = 5288 [38.8%]), Ireland (*n* = 168 [1.2%]), Italy (*n* = 3509 [25.7%]), The Netherlands (*n* = 1263 [9.3%]), Portugal (*n* = 108 [0.8%]), Spain (*n* = 838 [6.1%]), Switzerland (*n* = 156 [1.1%]) and United Kingdom (*n* = 698 [5.1%]) (Fig. 2).

**Table 1:** Baseline demographics and clinical characteristics of patients included in ETNA-AF-Europe

Characteristic	Total	Edoxaban 60 mg OD	Edoxaban 30 mg OD
Patients, N (%)	13,638 (100)	10,444 (76.6)	3194 (23.4)
Male, %	56.6	60.6	43.5
Age, years, mean (SD)	73.6 (9.52)	71.8 (9.23)	79.6 (7.87)
By age sub-groups, %			
< 65 years	15.4	18.8	4.1
65–74 years	33.7	38.5	18.0
75–84 years	40.3	37.3	50.4
≥ 85 years	10.6	5.4	27.5
Body weight, kg, mean (SD)	81.0 (17.34)	83.5 (16.77)	72.8 (16.63)
BMI, kg/m^2^, mean (SD)	28.1 (5.14)	28.6 (5.06)	26.5 (5.07)
SBP, mmHg, mean (SD)	133.4 (18.04)	133.7 (17.90)	132.6 (18.46)
DBP, mmHg, mean (SD)	78.3 (10.90)	79.0 (10.91)	76.2 (10.60)
Current smoking, %	6.3	6.9	4.4
Alcohol, %			
No	44.8	42.0	54.0
CrCl (reported), mL/min, mean (SD)	69.4 (24.23)	75.5 (22.65)	50.8 (18.90)
By CrCl subgroups, %			
< 15	0.6	0.7	0.2
(15, 30)	1.9	0.3	6.8
(30, 50)	18.0	6.7	52.9
(50, 80)	48.2	53.6	31.6
≥ 80	31.3	38.7	8.5
Patients with dose reduction criteria, %			
Body weight ≤ 60 kg, %	10.4	5.0	27.6
CrCl 15–50 mL/min, %	19.9	7.0	59.7
Concomitant use of P-gp inhibitors^a^, %	1.0	0.9	1.5
(calc.) CHADS_2_, mean (SD)	1.7 (1.07)	1.6 (1.05)	2.1 (1.03)
(calc.) CHA_2_DS_2_-VASc, mean (SD)	3.1 (1.40)	2.9 (1.37)	3.8 (1.26)
CHA_S_DS_2_-VASc score = 0, %	2.3	2.8	0.3
CHA_S_DS_2_-VASc score = 1, %	10.3	12.6	2.5
(calc.) HAS-BLED, mean	2.6 (1.13)	2.4 (1.11)	3.0 (1.10)
Frailty^b^, %			
Yes	10.6	6.1	25.3
No	82.7	87.3	67.4
Not known	6.7	6.6	7.3
Previous history of CV disease, %			
Hypertension	76.9	75.9	80.0
Congestive heart failure	5.8	4.7	9.5
Myocardial infarction	4.3	3.7	6.2
Angina pectoris	1.5	1.3	2.1
Valvular disease	17.7	16.2	22.9
Peripheral artery disease	3.3	2.8	5.2
Previous history of diabetes mellitus, %	21.9	20.5	26.7
Previous history of stroke and ICH, %			
Ischaemic stroke	5.9	5.6	6.9
Stroke, unknown	0.6	0.5	0.8
Transient ischaemic attack	3.3	3.2	3.7
Intracranial haemorrhage	0.5	0.4	0.6
Previous history of bleeding, %			
Any bleeding	3.1	2.5	5.2
GI bleeding (Major or CRNM)	0.8	0.5	1.7
Major	1.0	0.8	1.6
CRNM	1.0	0.8	1.8
Minor	1.1	0.9	1.8
Renal disease (including dialysis), %	27.0	19.5	51.6
Current AF type, %			
Paroxysmal	53.6	54.5	50.4
Persistent	24.4	25.3	21.2
Long-standing persistent	2.4	2.3	2.9
Permanent	19.6	17.8	25.5
Time since first AF diagnosis, months			
Mean (SD)	25.7 (46.88)	24.9 (47.01)	28.4 (46.37)
IQ, Q1–Q3	29.3–0.4	26.5–0.4	37.5–0.6
Previous use of AF relevant medication, %			
VKA	16.9	16.3	18.9
NOAC (other)	8.0	6.9	11.6
Antiarrrhythmics	5.0	5.0	4.8
Antiplatelet	15.1	14.8	16.2

^a^ P-gp inhibitors requiring edoxaban dose reduction: cyclosporine, dronedarone, erythromycin, ketoconazole

^b^ There was no specific definition for frailty; it was left to the discretion of the physician to categorise a patient as frail

*AF* atrial fibrillation, *BMI* body mass index, *CKD* chronic kidney disease, *CrCl* creatinine clearance, *CRNM* clinically relevant non-major, *CV* cardiovascular, *DBP* diastolic blood pressure, *GI* gastrointestinal, *ICH* intracranial haemorrhage, *IQ* interquartile range, *NOAC* non-vitamin K antagonist oral anticoagulant, *NSAIDs* non-steroidal anti-inflammatory drugs, *OD* once daily, *P-gp* P-glycoprotein, *SBP* systolic blood pressure, *SD* standard deviation, *VKA* vitamin K antagonist

**Fig. 3:**
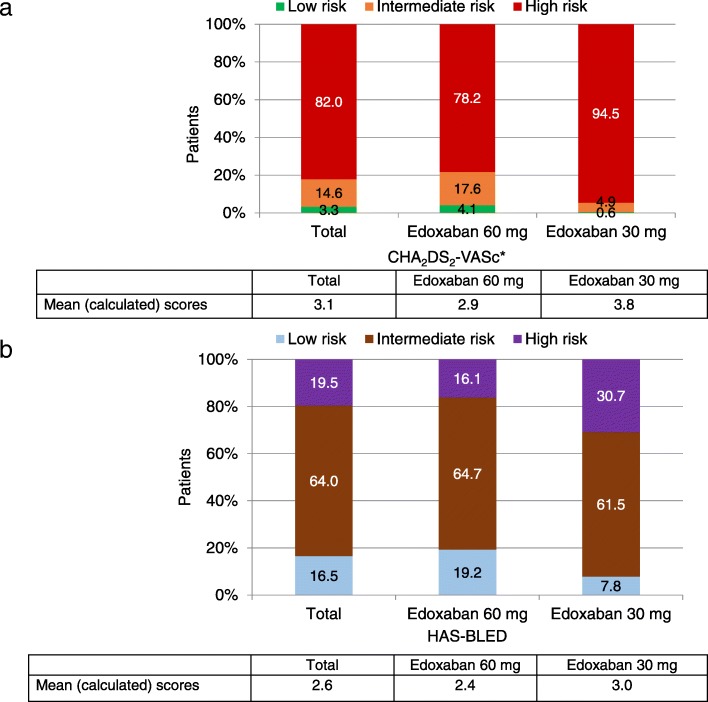
Mean CHA_2_DS_2_-VASc and HAS-BLED [calculated] score distribution by dose groups. **a** CHA_2_DS_2_-VASc score distribution^(7)^: Low risk: Score = 0 for men, 1 for women. Intermediate risk: Score = 1 for men, 2 for women. High risk: Score ≥ 2 for men, > 2 for women. **b** HAS-BLED score distribution: Low risk: Score < 2. Intermediate risk: Score 2–3. High risk: Score ≥ 4

The mean (SD) patient age was 73.6 ± 9.52 years (interquartile range Q1–Q3: 68.0–80.0 years) with an average creatinine clearance (CrCl) of 69.4 mL/min as calculated according to the Cockroft-Gault equation, and 7718 (56.6%) of the patients were male. The mean (SD) body weight was 81.0 ± 17.34 kg and mean (SD) body mass index was 28.1 ± 5.14 kg/m^2^. Mean CHA_2_DS_2_-VASc and HAS-BLED scores were 3.3 ± 1.45 and 1.9 ± 1.02 as reported by the investigators, respectively. In contrast, the calculated CHA_2_DS_2_-VASc and HAS-BLED mean scores were 3.1 ± 1.40 and 2.6 ± 1.13, respectively. Overall, 452 (3.3%) of patients had a CHA_2_DS_2_-VASc score of 0; 1997 (14.6%) of patients had a CHA_2_DS_2_-VASc of 1 and 11,186 (82.0%) of patients had a CHA_2_DS_2_-VASc score of ≥2 (defined according to ESC guidelines [7] (Fig. 3). The first diagnosis of AF had been made over two years before enrolment (mean [Q1–Q3]: 25.7 [29.3–0.4] months) (Table 1). Just over half of the patient population presented with paroxysmal AF, and 19.6% of the patients presented with permanent AF. Hypertension was the most common cardiovascular comorbidity (76.9%), followed by valvular heart disease (17.7%) and heart failure (5.8%) (Table 1). Patients with a previous ischaemic stroke accounted for 5.9%, transient ischaemic attack 3.3, and 4.3% had a prior myocardial infarction. A prior bleeding event was found in 3.1% of patients (Table 1).

**Fig. 4:**
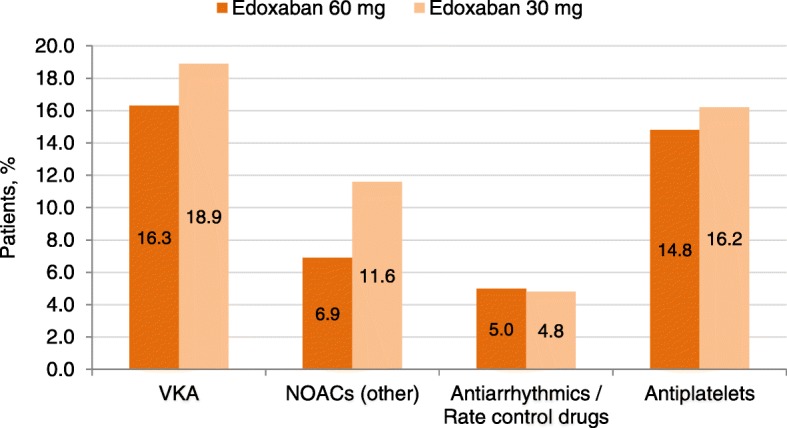
Percentage of patients who received antithrombotic or antiarrhythmic treatment prior to enrolment. AF, atrial fibrillation; BL, baseline; NOAC, non-vitamin K antagonist oral anticoagulant; VKA, vitamin K antagonist

Overall, 10,242 (75.1%) patients were not on anticoagulation prior to initiating edoxaban, whilst 2305 (16.9%) switched from a VKA (Fig. 4): 904 (39.2%) from warfarin, 732 (31.8%) from acenocumarol and 658 (28.5%) from phenprocoumon. Furthermore, 1091 (8.0%) switched from another NOAC: 341 (31.3%) from apixaban, 283 (25.9%) from dabigatran and 453 (41.5%) from rivaroxaban and 14 (1.3%) from others (not specified).

Of the 13,638 patients, 10,444 (76.6%) received edoxaban 60 mg and 3194 (23.4%) received 30 mg once daily. Adherence to the edoxaban SmPC was 83.8%, with 11,432/13,638 patients being dosed in line with the edoxaban label. Of the 2206 patients (16.2%) not dosed following the label, 1031 patients (7.5%) received 30 mg edoxaban without the criteria for dose reduction, whereas 1175 patients (8.6%) received edoxaban 60 mg even though at least one of the dose reduction criteria was present.

**Fig. 5:**
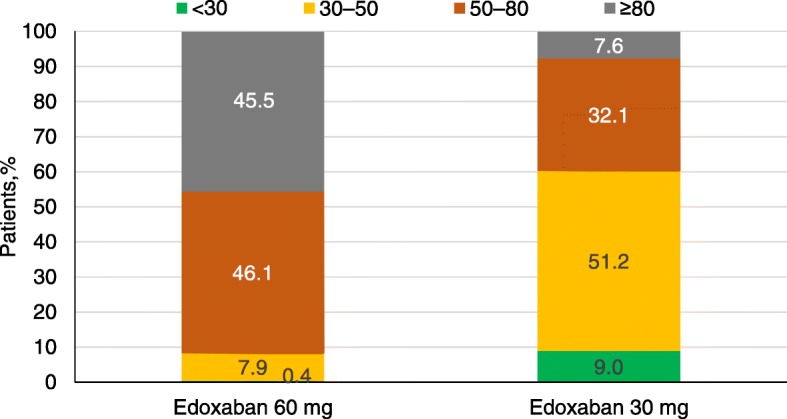
The stages of chronic kidney disease [calculated, Cockroft Gault] at baseline by dose groups

There were considerable differences in the characteristics of patients receiving the 60 mg vs the 30 mg dose. The mean baseline CrCl was 75.5 mL/min and 50.8 mL/min for the edoxaban 60 mg and 30 mg dose groups, respectively. 60.2% of patients in the edoxaban 30 mg dose group, compared to only 8.3% in the edoxaban 60 mg group, had a baseline CrCl of ≤50 ml/min (Fig. 5). A history of chronic kidney disease was reported in 19.5 and 51.6% of patients receiving edoxaban 60 mg and 30 mg doses, respectively.

**Fig. 6:**
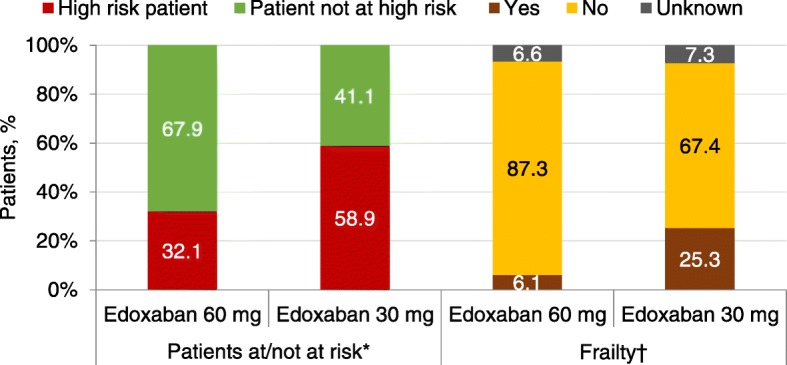
Difficult-to-treat patients at baseline categorised by dose groups. *A patient is considered as a high risk patient if he/she has at least one of the following: prior stroke, prior major bleeding, prior ICH or (calculated) CHA_2_DS_2_-VASc ≥4. ^†^There was no specific definition for frailty; it was left to the discretion of the physician to categorise a patient as frail

High-risk patients, defined in the ETNA-AF-Europe registry as patients with a high risk for either stroke or bleeding based on presence of either a prior stroke, prior major bleeding or prior ICH, or a CHA_2_DS_2_-VASc score ≥ 4, comprised 38.4% of the overall population; with 32.1 and 58.9% of those receiving edoxaban 60 mg and 30 mg doses, respectively categorised as high-risk patients (Fig. 6). Overall, 1442 (10.6%) of patients were considered frail, more than half of those were receiving edoxaban 30 mg once daily. There was no specific definition for frailty; it was left to the discretion of the physician to categorise a patient as frail.

A large majority of patients (77.8%) receiving edoxaban 30 mg versus 42.7% of patients on edoxaban 60 mg were aged ≥75 years. CHA_2_DS_2_-VASc scores ≥4 were more frequent in patients receiving edoxaban 30 mg (58.2%) versus edoxaban 60 mg dose (31%) (Fig. 3).

A greater proportion of patients with a previous history of CV disease received edoxaban 30 mg as compared with edoxaban 60 mg dose. Ischaemic stroke was reported in 6.9% versus 5.6% in the edoxaban 30 mg versus 60 mg dose group and myocardial infarction in 6.2% versus 3.7%, respectively (Table 1). Likewise, a history of any bleeding was documented more frequently for the edoxaban 30 mg versus edoxaban 60 mg dose (5.2% versus 2.5%, respectively).

## Discussion

The baseline characteristics of the ETNA-AF-Europe registry here reported indicate that among the 13,980 patients enrolled, there is a consistent percentage of elderly patients, with 50.9% of them ≥75 years of age. Most enrolled patients are naïve to prior anticoagulation (75.1%) and more than half have paroxysmal AF (53.6%). The use of the 60 mg full dose is overall predominant (76.6%), but among the high risk and frail patients, the use of 30 mg is higher (58.9 and 56%, respectively). For randomisation in ENGAGE AF-TIMI 48, a CHADS_2_ score of 2 or more was required, whereas the approved European Medicines Agency label requires only a CHADS_2_ score of 1 to be eligible for treatment with edoxaban. Daiichi Sankyo, the ETNA-AF steering committee and the European Medicines Agency have therefore jointly designed the ETNA-AF-Europe study to provide important additional safety information on the use of edoxaban in routine care.

The baseline demographics and clinical characteristics of patients receiving edoxaban in ETNA-AF-Europe were broadly similar to those enrolled in the European cohort of the ENGAGE AF-TIMI 48 trial, yet there were some notable differences (Table 2). The mean CHADS_2_ and CHA_2_DS_2_-VASc scores were considerably lower in the ETNA-AF-Europe registry compared with the ENGAGE AF-TIMI trial (CHADS_2_: 1.7 versus 2.8 and CHA_2_DS_2_-VASc: 3.1 versus 4.2, respectively) and patients more frequently presented with paroxysmal AF (53.6% versus 26.6%, respectively) (Table 2). The lower mean CHADS_2_ score in ETNA-AF-Europe likely reflected the difference between the European label of edoxaban (CHADS_2_ ≥ 1) and the inclusion criteria of ENGAGE AF-TIMI 48 (a score of 2 or higher on the CHADS_2_ risk assessment), and may also hint to a cautious approach of prescribing physicians to newly marketed medicines.

**Table 2:** Comparison of baseline characteristics of patients in the ETNA-AF-Europe registry and ENGAGE AF-TIMI 48 trial

	ETNA-AF-Europe *N* = 13,638	ENGAGE AF-TIMI 48^a^ Corresponding ETNA-AF countries^b^ *N* = 2123
Age (y), mean (SD)	73.6 (9.52)	72.7 (8.09)
By age sub-groups, n (%)		
< 65 years	2096 (15.4)	344 (16.2)
65–75 years	4598 (33.7)	764 (36.0)
≥ 75 years	6939 (50.9)	1015 (47.8)
Male, %	56.6	62.4
BMI (kg/m^2^), mean (SD)	28.1 (5.14)	29.8 (5.39)
Weight (kg), mean (SD)	81.0 (17.34)	85.4 (17.47)
eCrCl (mL/min), mean (SD)	69.4 (24.23)	75.5 (28.96)
CHADS_2_, mean (SD)	1.7 (1.07)	2.8 (0.93)
CHA_2_DS_2_-VASc, calculated, mean (SD)	3.1 (1.40)	4.2 (1.31)
HAS-BLED, mean (SD)	2.6 (1.13)	1.6 (0.92)
Hypertension, %	76.9	92.4
Diabetes, %	21.9	39.1
Myocardial infarction, %	4.3	2.9
Ischaemic stroke, %	5.9	15.5
Transient ischaemic attack, %	3.3	11.9
Congestive heart failure, %	5.8	48.2
Paroxysmal AF, %	53.6	26.6
Persistent AF, %	24.4	24.1
Permanent AF, %	19.6	49.4
Renal disease (including dialysis), %	27.0	11.9

^a^mITT

^b^Belgium, Switzerland, Germany, Spain, United Kingdom, Italy, Netherlands, Portugal

% values are based on non-missing data

*AF* atrial fibrillation, *BMI* body mass index, *CHADS*_*2*_ Congestive heart failure, Hypertension, Age ≥ 75 years, Diabetes mellitus, Stroke (double weight), *eCrCl* estimated creatinine clearance (Cockcroft-Gault), *ENGAGE AF-TIMI 48 study* Effective aNticoaGulAtion with Factor Xa next GEneration in Atrial Fibrillation-Thrombolysis In Myocardial Infarction study 48, *ETNA-AF* Edoxaban Treatment in routine cliNical prActice, *mITT* modified intent-to-treat, *SD* standard deviation

In keeping with the different inclusion criteria, cardiovascular comorbidities such as hypertension, congestive heart failure and history of ischaemic stroke were more common in patients in the European ENGAGE AF-TIMI 48 trial versus those included in ETNA-AF-Europe. Also, prior CV events such as ischaemic stroke and transient ischaemic attack were fewer in ETNA-AF-Europe versus the ENGAGE AF-TIMI 48 trial.

There are important markers of high risk of complications in the ETNA-AF-Europe population. Patients receiving edoxaban in routine care are slightly older than those enrolled in the ENGAGE AF-TIMI 48 trial. The population in the ETNA-AF-Europe analysis had a higher bleeding risk than those recorded in the corresponding countries in ENGAGE AF-TIMI 48, with mean HAS-BLED scores of 2.5 and 1.6 in ETNA-AF-Europe and ENGAGE AF-TIMI 48, respectively (Table 2). The ETNA-AF-Europe population shows a slightly lower creatinine clearance and a higher proportion of patients with chronic kidney disease (Table 2). These findings underpin the general assumption that patients at a higher risk are under-represented in phase III trials, including those of anticoagulants for stroke prevention in AF.

In ETNA-AF-Europe, the mean investigator-reported CHA_2_DS_2_-VASc score was higher than the formally calculated score whereas the mean investigator reported HAS-BLED score was lower than the formally calculated score. These findings suggest that physicians may have overestimated the risk of stroke and underestimated the risk of bleeding in the ETNA-AF-Europe population. Specifically, the HAS-BLED score may potentially not be widely used in daily clinical practice so the physicians’ lack of familiarity with this score could have played a role in the underestimation of bleeding risk. Further investigations in the ETNA-AF-Europe database for the potential reasons related to a wrong perception of risks such as consideration of specific comorbidities or patient history may bring more clarity to this finding. For now, this finding highlights the continued need for educating health care professionals and patients in Europe concerning anticoagulation [22].

In routine clinical practice, it is commonly observed that NOACs are frequently prescribed at a lower dose despite not meeting the dose reduction criteria listed in the label [23, 24]. Therefore, compared with the pivotal clinical trials, the use of reduced NOAC doses in daily clinical practice appears to be more frequent [25, 26]. A retrospective study reported the use of a reduced dose of NOACs in 56.8% of patients with no clear indication, and use of non-reduced dose in 43.2% of patients while indicated [25]. Such findings suggest the need to strengthen dosing education of NOACs in clinical practice. Notably, the distribution of the edoxaban 60 mg and 30 mg dosing in ETNA-AF-Europe was largely in line with the distribution within corresponding countries from ENGAGE AF-TIMI 48; a total of 23.4% of patients in ETNA-AF-Europe received the reduced dose of edoxaban 30 mg which was similar to the percentage of patients (21.8%) eligible for dose reduction in the European cohort of the ENGAGE AF-TIMI 48 trial.

Among the edoxaban dose reduction criteria listed in the SmPC, reduced renal clearance ≤50 ml/min is by far the most frequently applied criterion in a Western population [27], whereas low body weight (below 60 kg) or concomitant use of certain P-gp-inhibitors are less often met. The ETNA programme will provide information on patients characteristics in other parts of the world, where weight < 60 kg may be more common.

Since poor renal function is associated with old age and various comorbidities, the differences in baseline characteristics of patients receiving edoxaban 30 mg and 60 mg groups are broadly in line with expectations. History of congestive heart failure, myocardial infarction, angina pectoris and valvular heart disease was more commonly observed in the 30 mg dose group as compared with the patients receiving the 60 mg dose (Table 1). High risk patients, predefined as those with prior stroke, prior major bleed, prior intracranial haemorrhage, or CHA_2_DS_2_-VASc score ≥ 4 accounted for 38.4% of patients in ETNA-AF and a higher proportion of these difficult-to-treat patients received the edoxaban 30 mg dose versus edoxaban 60 mg dose in ETNA-AF-Europe. The same was true for patients considered frail by the investigator.

In ETNA-AF-Europe, a high adherence of 83.8% to the dose selection criteria according to the SmPC was observed. The consistent dosing criteria for edoxaban across all indications may have contributed to this finding. Nevertheless, not all patients were dosed according to the label; approximately 16% of patients received a dose that was not in line with the SmPC, with 7.5% (*n* = 1031) of patients receiving edoxaban 30 mg instead of the recommended 60 mg, and 8.6% (*n* = 1175) of patients receiving 60 mg instead of the recommended 30 mg. The rationale for prescribing the 60 mg dose despite the presence of a dose reduction criterion is currently unclear and needs further analyses. The general overestimation of the CHA_2_DS_2_-VASc score combined with the underestimation of the HAS-BLED score by the ETNA-AF-Europe investigators or unavailability of renal function at the time of prescribing, may both have played a role in this context. Future analyses on specific characteristics of these patients such as very high body weight or certain co-morbidities are warranted. Furthermore, patient age, creatinine clearance or body weight close to the indicated dose reduction threshold, or the fear of iatrogenic bleeding in a fragile patient population that already had a frequent history of bleeding may have contributed to the choice of the lower edoxaban dose deviating from the label. In support, perceived frailty of patients was more frequently reported in patients receiving the 30 mg dose (Fig. 6), which could have influenced the prescribing pattern independent of the criteria in the label. Publications such as the 2018 European Heart Rhythm Association Practical Guide [28] have certainly helped in raising awareness regarding the need for appropriate dosing of NOACs. The details in the EHRA guide differ somewhat from the criteria outlined in the SmPC. It will be interesting to apply these additional criteria to the ETNA-AF-Europe population and this study provides an opportunity to look into this.

It is widely accepted that randomised trials are the gold standard for ascertaining the efficacy and safety of a given therapy. However, RCTs are not fully representative of an unselected real-world population due to their highly controlled settings. For example, in all phase 3 AF NOAC trials, patients with very high bleeding risk were largely excluded, resulting in paucity of data on these patients. In addition, the patient population specified in the label is usually broader than the key inclusion criteria of the pivotal trials. On the other hand, a prospective registry might also have limitations, due to the lack of randomisation: physicians might tend to prescribe a certain therapy to a specific category of patients, and furthermore patients could opt in for enrolment. These features might cause a selection bias that may hamper the generalizability of the observed results.

In our registry, the majority of AF patients were anticoagulation-naïve prior to the initiation of edoxaban. Patients were also reported to switch from another NOAC to edoxaban, illustrating a clinical need for more than one NOAC for stroke prevention in AF. The recent registry data have shown that 95% of edoxaban users enrolled via the Danish National Prescription Registry [16] had previously received some anticoagulant treatment, with 77% switching directly from another anticoagulant treatment to edoxaban (45% from VKA and 32% from other NOACs). In this registry, users of edoxaban were comparable with users of other NOACs, with a median age of 75 versus 72–76 years and 57% versus 53–59% males. These baseline results are the first consistent evidence of real-world use of once daily edoxaban, the most recently approved NOAC, in Europe.

## Conclusion

Edoxaban is predominantly used in routine clinical practice for older AF patients, mostly anticoagulation-naïve with dosing in line with recommendations in the approved European Union label for a high proportion of patients (84%). The patient population enrolled in ETNA-AF-Europe is similar to those enrolled in the ENGAGE AF TIMI-48 trial and showed a similar proportional usage of the reduced 30 mg edoxaban dose. Differences between the populations include slightly higher age, more patients with paroxysmal AF, lower CHA_2_DS_2_-VASc score, higher bleeding risk, less cardiovascular comorbidities and more renal impairment in ETNA-AF-Europe.

## Data Availability

The data that support the findings of this study are available from Daiichi Sankyo but restrictions apply to the availability of these data, which were used under license for the current study, and so are not publicly available. Data are however available from the authors upon reasonable request and with permission of Daiichi Sankyo.

## Abbreviations

AF: Atrial fibrillation
CrCl: Creatinine clearance
CV: Cardiovascular
ETNA-AF-Europe: Edoxaban treatment in routine clinical practice for patients with non valvular atrial fibrillation
NOACs: Non-vitamin K antagonist (VKA) oral anticoagulants
PRAC: Pharmacovigilance risk assessment committee
RCTs: Randomised controlled trials
RWE: Real world evidence
SmPC: Summary of product characteristics
VTE: Venous thromboembolism
